# Posterior tibial artery aneurysm: a case report with review of literature

**DOI:** 10.1186/1471-2482-14-37

**Published:** 2014-06-16

**Authors:** Jayesh Sagar, Mathew Button

**Affiliations:** 1 Department of Vascular Surgery, Royal Sussex County Hospital, Brighton BN1 2HS, UK

## Abstract

**Background:**

Aneurysms infra-patellar region are uncommon. Of them, true aneurysms are very rare and that of posterior tibial artery are extremely rare. The more common, pseudoaneurysms are commonly associated with trauma whereas the true ones are linked with either inflammatory or mycotic origins.

**Case Presentation:**

We reported another case of true aneurysm of posterior tibial artery without any evident aetiology. This was repaired with resection of aneurysm followed by interposition vein graft.

**Conclusion:**

Through this report, we discussed the rarity, review of literature and management of this unusual condition.

## Background

Aneurysm is one of the common conditions that any vascular surgeon comes across frequently. Aneurysms are more common in the proximal arteries such as femoral and popliteal arteries compared to distal small blood vessels and their management options are well documented in English literature. However it is rare in the infra-popliteal region. Of them, false aneurysms are more common and are usually associated with trauma [1-5]. The true aneurysms of infrapopliteal region are extremely rare and most of them have been reported to be associated with either infection or inflammatory process [6-11]. Of the infra-popliteal blood vessels, the true dilatation of posterior tibial artery has been reported in the single digit numbers [12-14]. To add, we reported another case of true aneurysm of posterior tibial artery. We believe this to be the twelfth of such case reports in English literature. Along with its rarity, we also discussed the literature and its management options.

## Case presentation

**Figure 1:**
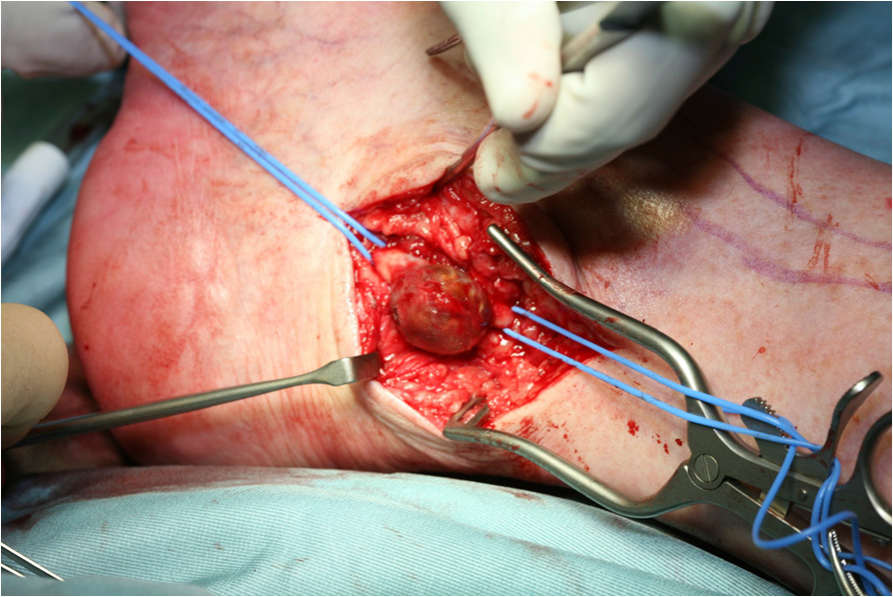
Exposed posterior tibial artery aneurysm.

**Figure 2:**
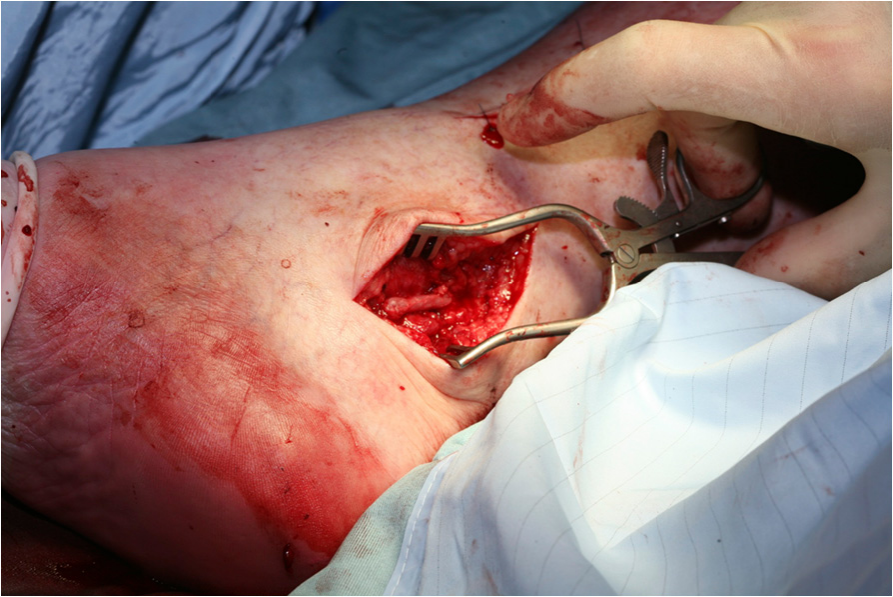
Vein graft repair of posterior tibial artery aneurysm.

**Figure 3:**
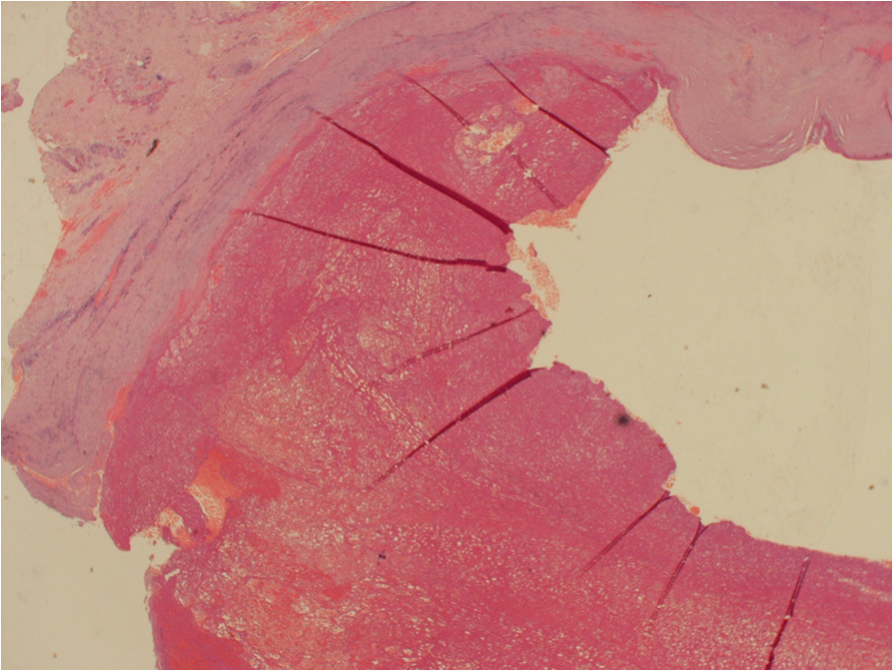
Thrombus adherent to endothelial surface.

**Figure 4:**
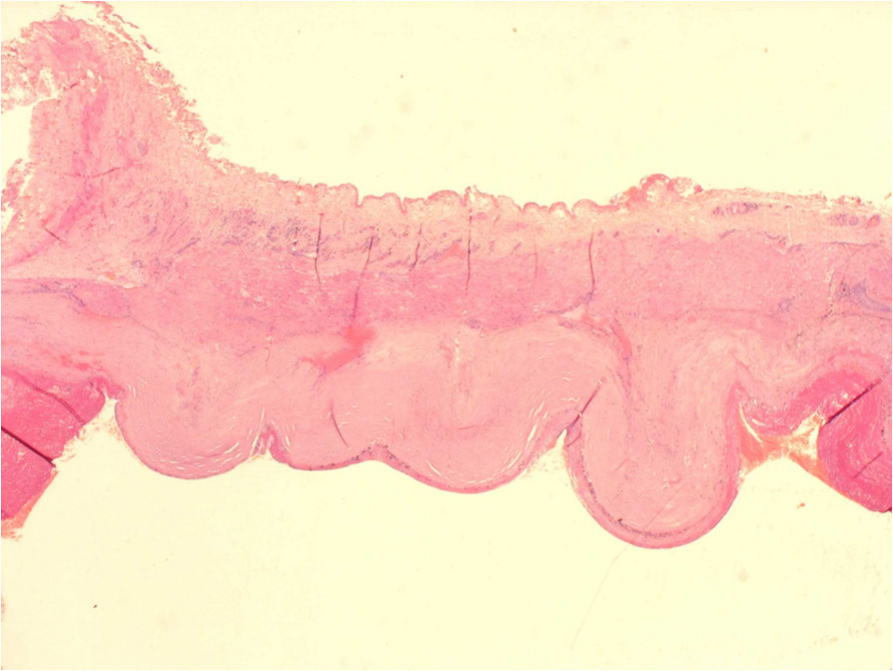
Haematoxylin and Elastin staining of aneurysmal wall.

**Figure 5:**
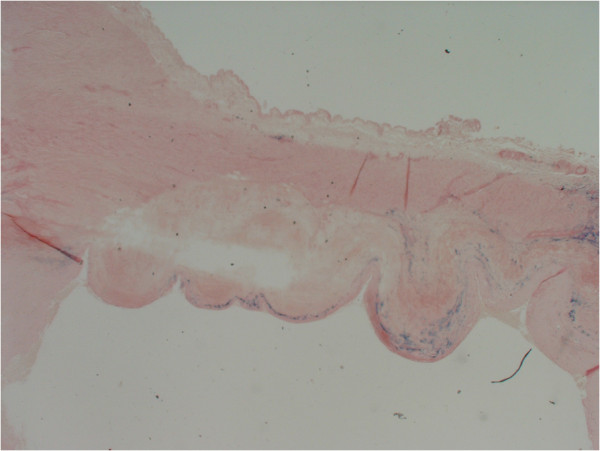
Perl’s staining for Iron in aneurysmal wall (Purple staining).

**Figure 6:**
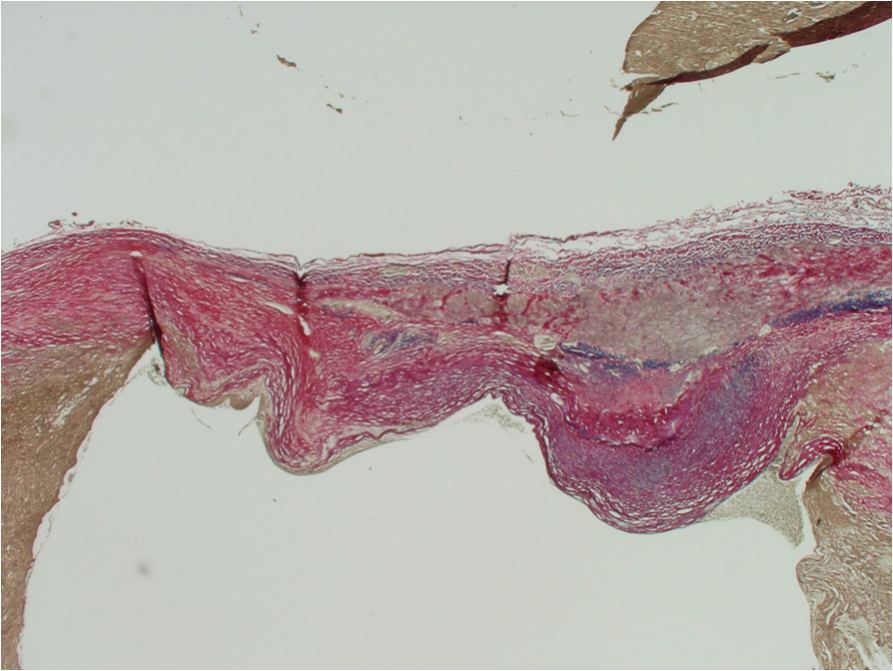
Elastic Van Gieson staining of aneurysmal wall.

A 64-year old white English male was presented to the vascular outpatient clinic with history of a lump behind his right ankle on inner aspect over a year. Apart from the presence of a lump, he denied any other complaints including any violent or repeated trauma. He also suffered from diet controlled Type II diabetes mallitus and gout and had tonsillectomy and manipulation of colles’ fracture in childhood. He was not taking any antiplatelet or anti-coagulation medications and denied any use of tobacco. On examination, there was 3 × 3 cm size lump just behind the right medial malleous which was non-tender and non-pulsatile. The peripheral pulses were easily palpable on either side. There was no evidence of aneurysm anywhere else in the body on clinical examination. He underwent Doppler ultrasound which confirmed 1.4 cm size aneurysm of posterior tibial artery with presence of mural thrombus. The distal and proximal parts of posterior tibial artery, anterior tibial artery and popliteal artery were reported normal. He underwent an elective operation in the form of excision of aneurysm (Figure 1) followed by reversed vein graft from the same leg (Figure 2). Histology confirmed the true aneurysm of posterior tibial artery with mural thrombus attached to the intima of the vessel with normal arterial wall pattern without any evidence of connective tissue disorders, arteritis, necrotizing vasculitis, infection or inflammation (Figures 3, 4, 5 and 6). The bacteriology examination neither revealed any organisms nor grown any organisms in the culture media. He recovered well postoperatively and was discharged the next day. At follow up at one year, he did not develop any complications and colour Doppler revealed patent posterior tibial artery.

### Discussion

False aneurysms are more common in comparison to the true aneurysms of infrapatellar blood vessels. There have been only very few case reports of true aneurysms of posterior tibial artery published [13,14]. The precise aetiological factors are not identified, but trauma, collagen vascular pathology, fibromuscular dysplasia, inflammation, infection and atherosclerosis were suggested.

The most common clinical presentations include asysmptomatic lump, distal embolism and aneurysm thrombosis. Paraesthesia secondary to such aneurysm is rare but reported in literature [13]. Tshomba et al. reported 9% of cases presented with distal critical ischaemia of which two third ended up having midfood amputation, while only 3% of cases presented with rupture that resulted in acute compartment syndrome [13]. Differential diagnoses of this aneurysm include tendon cyst, neurinoma, soft tissue tumour or pulsatile masses [15].

In our case, we could not find any aetiological factor for the aneurysm development. Out of eleven published case reports of true posterior tibial artery aneurysms, in four of them, aetiology was unknown [16,17,12,9]. Two cases were reported secondary to degenerative changes [18,13] and another three cases were secondary to mycotic infection [6,11]. In one case, polyarteritis nodosa was responsible for such aneurysm [19] while in another case, histology was suggestive of syphilitic infection but immunostaining and culture isolation did not confirm the diagnosis [14].

The management options vary from conservative approach to surgical excision followed by reconstitution of posterior tibial artery. Due to very limited number of published cases, a standard treatment has not been defined. Therefore, the indications for treating these lesions are still a matter of debate, but symptomatic aneurysms, asymptomatic large aneurysms and those with laminated thrombus should be offered treatment [20]. Yao and McCarthy observed asymptomatic aneurysm for seven years without any enlargement of aneurysm or any development of symptoms [12]. Borozan also reported and suggested conservative approach in asymptomatic aneurysms [21]. Although ligation of posterior tibial artery may be performed, especially in emergency settings, surgical excision with posterior tibial artery reconstitution either by primary repair or by interposition vein graft is the preferred treatment. Endovascular embolisation and percutaneous occlusion of aneurysm with various modalities are more commonly used in pseudo-aneurysms and are associated with risk of limb ischaemia.

In this case, patient underwent surgical excision followed by interposition vein graft. Out of eleven published case reports, six patients had undergone surgical excision of posterior tibial artery aneurysm with interposition vein graft [9,18,6,14,11], while four patients had ligation of posterior tibial artery [16,12,19,17]. One patient had surgical excision with primary end to end anastomosis of posterior tibial artery [13]. In our patient, anterior tibial artery was intact and one might question the need for operation in this report, however we believe that infrapopliteal aneurysms should be treated irrespective of symptomatology due to the risk of embolization, thrombosis and rupture leading to potential ischaemia and amputation.

## Conclusion

Although it is only twelfth reported case of true posterior tibial artery aneurysm, we recommend early diagnosis and treatment in the form of excision before it becomes symptomatic and complicated.

## Consent

Patient’s formal written consent was obtained for publication of this case report.

## Competing interests

The authors declare that they have no competing interests.

## Authors’ contributions

JS-Involved in the care of patient and has written the manuscript. MB-Involved in the care of patient and supervised the manuscript. Both authors read and approved the final manuscript.

## Pre-publication history

The pre-publication history for this paper can be accessed here:

http://www.biomedcentral.com/1471-2482/14/37/prepub
